# The Capacity to Secrete Insulin Is Dose-Dependent to Extremely High Glucose Concentrations: A Key Role for Adenylyl Cyclase

**DOI:** 10.3390/metabo11060401

**Published:** 2021-06-19

**Authors:** Katherine M. Gerber, Nicholas B. Whitticar, Daniel R. Rochester, Kathryn L. Corbin, William J. Koch, Craig S. Nunemaker

**Affiliations:** 1 Translational Health, Honors Tutorial College, Ohio University, Athens, OH 45701, USA; kg010716@ohio.edu; 2 Biomedical Sciences, Heritage College of Osteopathic Medicine, Ohio University, Athens, OH 45701, USA; nw575612@ohio.edu (N.B.W.); dr935216@ohio.edu (D.R.R.); corbink1@ohio.edu (K.L.C.); wk922015@ohio.edu (W.J.K.); 3 Translational Biomedical Sciences Program, Heritage College of Osteopathic Medicine, Ohio University, Athens, OH 45701, USA; 4 Diabetes Institute, Heritage College of Osteopathic Medicine, Ohio University, Athens, OH 45701, USA

**Keywords:** amplifying pathway, hyperglycemia, adenylyl cyclase, incretins, glucokinase, forskolin, cAMP, exenatide, diabetes, insulin, islets

## Abstract

Insulin secretion is widely thought to be maximally stimulated in glucose concentrations of 16.7-to-30 mM (300-to-540 mg/dL). However, insulin secretion is seldom tested in hyperglycemia exceeding these levels despite the Guinness World Record being 147.6 mM (2656 mg/dL). We investigated how islets respond to 1-h exposure to glucose approaching this record. Insulin secretion from human islets at 12 mM glucose intervals dose-dependently increased until at least 72 mM glucose. Murine islets in 84 mM glucose secreted nearly double the insulin as in 24 mM (*p* < 0.001). Intracellular calcium was maximally stimulated in 24 mM glucose despite a further doubling of insulin secretion in higher glucose, implying that insulin secretion above 24 mM occurs through amplifying pathway(s). Increased osmolarity of 425-mOsm had no effect on insulin secretion (1-h exposure) or viability (48-h exposure) in murine islets. Murine islets in 24 mM glucose treated with a glucokinase activator secreted as much insulin as islets in 84 mM glucose, indicating that glycolytic capacity exists above 24 mM. Using an incretin mimetic and an adenylyl cyclase activator in 24 mM glucose enhanced insulin secretion above that observed in 84 mM glucose while adenylyl cyclase inhibitor reduced stimulatory effects. These results highlight the underestimated ability of islets to secrete insulin proportionally to extreme hyperglycemia through adenylyl cyclase activity.

## 1. Introduction

Pancreatic beta cells secrete insulin in response to glucose stimulation to maintain blood glucose levels within a relatively narrow range [1]. Insulin is required to transport glucose from the bloodstream to target tissues. High blood glucose levels in the body are caused by problems with insulin secretion, insulin action, or both. Extremely high levels of glucose lead to the presentation of ketoacidosis or hyperosmolar hyperglycemic nonketoic syndrome, which are key indicators of the metabolic disease diabetes [2]. Poor control of glucose regulation in this disease can bring potential stupor, coma, or death [3].

In humans, insulin secretion is typically stimulated by glucose concentrations ranging from 4.4 to 6.6 mM (80–120 mg/dL) [2,4]. Moreover, it is generally accepted that the effective concentration of glucose for half of the maximal insulin secretion (EC_50_) is approximately 5 mM. These EC_50_ estimations are based on dose-response curves with the highest stimulation typically being described at 16.7 (300 mg/dL) to 30 mM (540 mg/dL). However, close inspection of published glucose dose-response curves suggests that even though a classic sigmoid dose-response curve should flatten out, the curves typically show an increasing trend of higher insulin secretion near the maximal glucose level tested. This provokes the hypothesis that insulin secretion may be sensitive to a much wider range of glucose concentrations than commonly thought [5,6,7,8,9].

Interestingly, there are multiple case reports of individuals who have had blood glucose levels greater than 100 mM (1800 mg/dL) and survived [2,4,10,11]. This includes the world record blood glucose level of 147.6 mM (2656 mg/dL) which was set by a young boy when admitted to the hospital [11]. The pathways involving insulin secretion at extremely high glucose concentrations have not been examined to our knowledge.

Glucose is the primary stimulator of insulin release from pancreatic beta cells. The ability of glucose to elicit an increase in intracellular calcium leading to insulin secretion is known as the triggering pathway [12,13,14]. This pathway begins when glucose enters the beta cell through both the GLUT 1 and GLUT 3 glucose transporters in human islets and the GLUT 2 transporter in mouse islets [13,14]. Glucose is then phosphorylated by glucokinase and yields glucose-6-phosphate which travels through glycolysis to yield pyruvate and ATP. Glycolysis and downstream mitochondrial metabolism drive the ratio of ATP to ADP to rise, leading to the closure of K_ATP_ channels. This closure activates the voltage-dependent calcium channels to allow the influx of calcium that constitutes the triggering pathway for insulin secretion.

In addition to the triggering pathway, many different intermediate metabolites of glucose and other cellular components are thought to participate in a series of events known as the amplification pathway(s) in which K_ATP_ channel closure is not the source of the increased insulin secretion. A review on this topic contained within this special issue describes this pathway as “the sequence of events that enables the secretory response to a nutrient secretagogue to exceed the secretory response of a purely depolarizing stimulus” [15]. Cyclic adenosine monophosphate (cAMP) is a powerful player in the amplification pathway-related insulin secretion. Glucose is a known factor leading to the upregulation of cAMP which is formed from ATP via adenylyl cyclases [16], although the exact mechanism of metabolism stimulated cAMP is unknown. Additionally, there is a glucose-linked amplification pathway that augments insulin secretion through adenylyl cyclase (AC) activation caused by incretin stimulation. Incretins are hormones secreted by endocrine cells in the small intestine after meal ingestion that lead to insulin secretion [12]. Gastric inhibitory polypeptide (GIP) and glucagon-like peptide-1 (GLP-1) are specific incretins that act on the beta cell. Incretins interact with G-protein-coupled receptors at the cell membrane and lead to an upregulation of cAMP [17,18]. The common theme is that cAMP is involved in the early steps leading to insulin secretion in these amplifying pathway(s).

In this study, we aim to determine the relationship between glucose stimulation and insulin secretion in a much higher glucose range than typically examined. We show that insulin secretion is maintained in response to extreme glucose, osmolarity does not affect insulin secretion or islet viability at extreme levels, and that intracellular calcium is maximized at 24 mM glucose. We also sought to identity possible pathway(s) used in extreme glucose conditions and found that adenylyl cyclase plays an important role.

## 2. Results

### 2.1. Insulin Secretion Is Maintained in Response to Extreme Glucose

**Figure 1:**
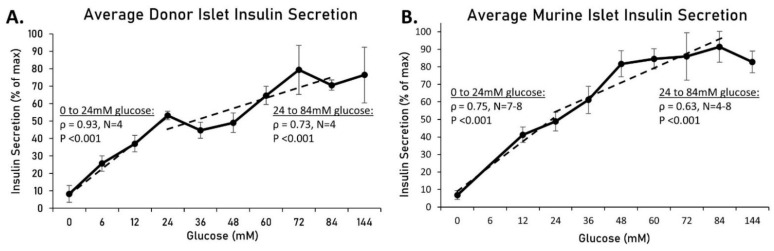
Human and murine islet insulin secretion in conditions of extremely high glucose. Islets from human donors (**A**) and mice (**B**) were placed in mannitol balanced KRB solutions containing glucose ranging from 0 mM to 144 mM for 1 h. Insulin secretion from each donor or mouse replicate was normalized to their respective maximal value and averaged. Dotted linear trendlines are drawn for 0–24 mM and 24–84 mM glucose to indicate slope, Spearman’s rho, and the respective *p*-value.

Identifying the maximal physiological response of biological systems often gives insight into function. We wanted to determine what happens to insulin secretion above the commonly accepted maximum concentration of glucose for stimulation. We thus examined insulin secretion from human and murine islets in conditions of extremely high glucose. Islets from human donors were placed in mannitol-balanced Krebs-Ring buffer (KRB) solutions containing glucose ranging from 0 mM to 144 mM for 1 h. As shown in Figure 1A, insulin secretion from each of the four donors was normalized to their respective maximal value and averaged amongst donors (see Table 1 for donor information). From 0 to 24 mM, there was a significant upward trend (rho = 0.93, *p* < 0.001) and from 24–84 mM, there was still a positive correlation, though not as strong (rho = 0.73, *p* < 0.001). Insulin secretion from murine islets was also measured (Figure 1B). From 0–24 mM, there was a strong upward trend (rho = 0.75, *p* < 0.001), and from 24–84 mM, there was still a significant positive trend (rho = 0.63, *p* < 0.001). Overall, there was a positive correlation between insulin and glucose for 0–24 mM, as expected, but a similar relationship exists from 24 mM glucose and beyond.

### 2.2. Increased Osmolarity Does Not Alter Insulin Secretion or Islet Viability

**Figure 2:**
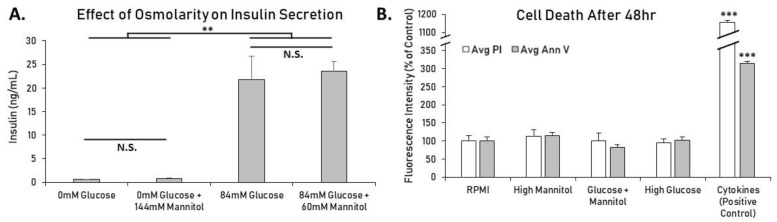
Effects of high glucose and mannitol on mouse islet insulin secretion and cell death. (**A**) Mouse islets were placed in wells containing modified KRB with 0 mM or 84 mM glucose ± mannitol balanced to 144 mM total for 1h. Increasing osmolarity using mannitol did not significantly alter insulin secretion in glucose-free or high-glucose solutions. Twenty islets per condition in duplicate ran in 2 separate trials (N = 4). (**B**) Islets were placed in either standard RPMI, high glucose (144 mM), high mannitol (144 mM), or both glucose (60 mM) and mannitol (84 mM). Islets treated overnight with 5 ng/mL IL-1beta and 10 ng/mL TNF-alpha were used as a positive control to show typical fluorescence levels of induced cell death. Islets in each condition ranged from N = 31–37 after combining two separate trials. All data are presented as mean ± SEM. ** *p* < 0.01, *** *p* < 0.001, N.S. = Not Significant.

To control for osmolarity as a variable, solutions were balanced to ~425 mOsm/L using the sugar alcohol mannitol. Mannitol was used to account for the difference in osmolarity between low and high glucose conditions, and here the effect of mannitol on its own was explored. Mannitol is not metabolized and therefore should not increase intracellular calcium or insulin secretion, although indirect factors such as membrane potential or ion flux might alter insulin secretion [19]. To investigate this possibility, islets were treated for 1 h in modified KRB with 0 mM glucose or 84 mM glucose with and without mannitol balanced to 144 mM. While it is known that various osmotic receptors on beta-cells have been shown to alter insulin secretion [20], as shown in Figure 2A, large increases in osmolarity caused no significant change in insulin secretion, and mannitol alone did not have a stimulatory effect. As expected, islets in each 84 mM glucose group secreted significantly more insulin (*p* < 0.01) than islets in each glucose-free group, but osmolarity changes due to mannitol did not significantly affect insulin secretion.

Next, we measured cell death using fluorescent microscopy to check for toxicity. Isolated mouse islets treated in standard RPMI, high glucose (144 mM), high osmolarity (144 mM), or a combination of both (84 mM glucose + 60 mM mannitol) for 48 h displayed no significant differences in cell death (propidium iodide) or apoptosis (annexin V) (Figure 2B). Proinflammatory cytokines were used as a positive control to induce beta-cell death. Collectively, these results indicate that substantial increases in osmolarity have no effect on cellular function or viability in mouse islets in these conditions.

### 2.3. Intracellular Calcium Is Maximally Stimulated in 24 mM Glucose

**Figure 3:**
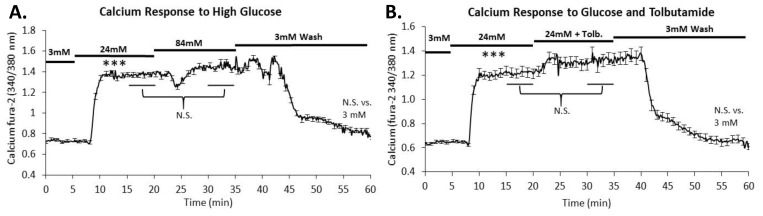
Effects of high glucose on intracellular calcium. (**A**) Intracellular calcium was measured in islets exposed to the increasing glucose conditions noted by the horizontal bars. Calcium levels increased sharply from 3 mM to 24 mM glucose but did not increase further when exposed to 84 mM glucose. Average calcium levels from 23 islets were calculated from the 15–20- and 30–35 min time points and showed no significant difference. (**B**) Intracellular calcium was measured in islets exposed to the increasing glucose conditions and tolbutamide noted by the horizontal bars. Calcium levels increased sharply from 3 mM to 24 mM but did not increase further when exposed to 24 mM glucose combined with 250 μM tolbutamide. Average calcium levels from 23 islets were calculated from the 15–20 and 30–35 min time points and showed no significant difference. *** *p* < 0.001 difference between 3 and 24 mM glucose.

To determine whether these responses were calcium dependent, mouse islets were loaded with the calcium probe fura-2AM and exposed to increasing stimulation as indicated by the black bars in Figure 3. As expected, a large increase was observed in response to 24 mM glucose stimulation. When the stimulus was increased from 24 to 84 mM glucose, average calcium levels of all islets were not significantly different (Figure 3A). Large increases in glucose above 24 mM do not appear to impact calcium handling. Furthermore, there was no significant difference between calcium levels in the 3 mM and the 3 mM wash (*p* > 0.05) showing that the extreme glucose did not alter the islet’s ability to control the secretion process.

Tolbutamide, a sulfonylurea known to depolarize beta cells via reduction of potassium permeability, leads to the opening of voltage-dependent calcium channels to subsequently trigger insulin release [21]. As anticipated, there was no significant difference (*p* > 0.05) between 24 mM and 24 mM combined with 250 µM tolbutamide in terms of intracellular calcium influx (Figure 3B). This supports the observation that intracellular calcium is maximally stimulated in 24 mM glucose.

### 2.4. Glycolytic Capacity to Secrete Insulin Dose-Dependently Extends above 24 mM Glucose

**Figure 4:**
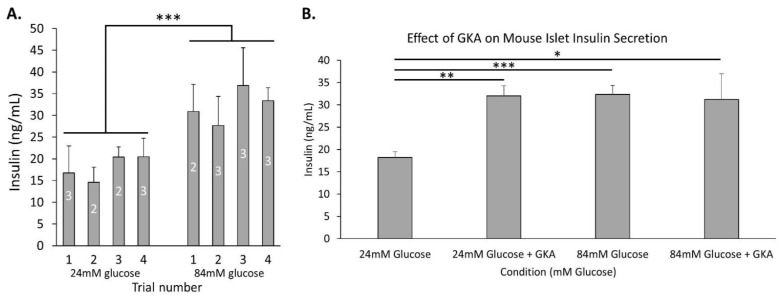
Insulin secretion in extremely high glucose is associated with increased glycolysis. (**A**) Insulin secretion was measured after 1h incubation in 24 mM glucose (regarded as maximal) and 84 mM glucose (regarded as extreme) shown for four separate trials with N = 2–3 replicates to demonstrate consistency of the difference in insulin secretion from trial to trial. Combining these trials, the data were significantly different with a *p*-value of <0.001 by two-tailed T-test between 24 and 84 mM glucose. (**B**) Mouse islets were placed in wells containing modified KRB with 24 mM or 84 mM glucose ± mannitol balanced to 84 mM total for 1 h with or without 500 nM GKA. The GKA increased insulin secretion significantly in 24 mM glucose (*p* < 0.01) but failed to do so in 84 mM glucose. All data are presented as ± SEM. N = 3–11 replicates. * *p* < 0.05, ** *p* < 0.01, *** *p* < 0.001.

**Figure 5:**
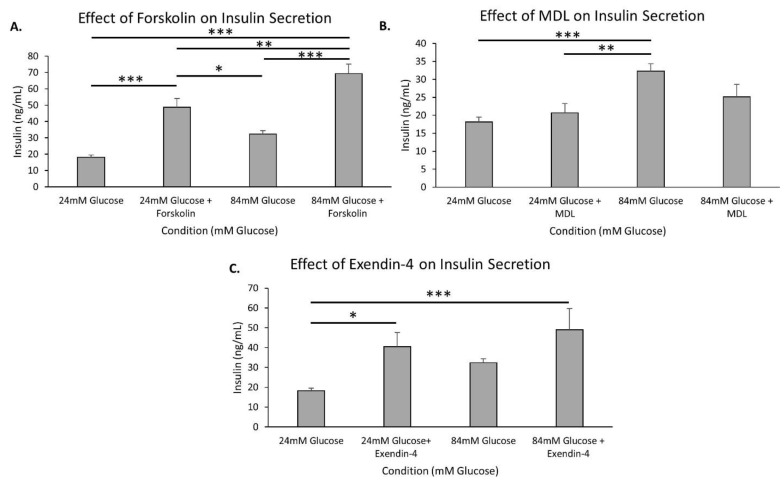
cAMP-related drug effect on insulin secretion in high glucose. (**A**–**C**) Murine islets were placed in wells containing modified KRB with 24 mM or 84 mM glucose ± mannitol balanced to 84 mM total for 1 h and then various drugs were added to each condition for 1 h of incubation before insulin was measured: 10 µM forskolin (**A**), 10 µM MDL 12,330A (**B**), and 10 nM exendin-4 (**C**) All data are presented as ± SEM. N = 5–11 replicates. * *p* < 0.05, ** *p* < 0.01, *** *p* < 0.001.

We next conducted a series of trials to determine what drives increased insulin secretion in high glucose. The following studies were all conducted in 24 mM glucose (regarded as maximal) and 84 mM glucose (regarded as extreme). As shown in Figure 4A, insulin secretion was significantly higher in 84 mM glucose compared to 24 mM glucose after combining all trials (*p* < 0.001). The averages are reproduced for comparisons made in Figure 4B and Figure 5.

To examine the contribution of glycolytic activity leading to insulin secretion, the glucokinase activator R0-28-1675 (hereafter called GKA) was utilized, and insulin measurements were taken. As shown in Figure 4B, islets treated with GKA in 24 mM glucose displayed a significant increase in insulin secretion compared to islets in 24 mM alone (*p* < 0.01). Insulin secretion in 84 mM glucose was on par with islets given GKA in 24 mM glucose, but GKA did not provide any additional stimulation in 84 mM glucose. Together, these data indicate that glycolytically driven insulin secretion continues to dose-dependently increase, nearly doubling insulin secretion between 24 and 84 mM glucose.

### 2.5. The cAMP Pathway Provides Additional Capacity for Insulin Secretion in Extremely High Glucose

cAMP, a known amplifier of insulin secretion, was examined by the use of forskolin, an adenylyl cyclase activator that increases cAMP levels. As shown in Figure 5A, in 24 mM glucose for 1 h, forskolin increased insulin secretion dramatically compared to the control 24 mM glucose (*p* < 0.001). There was also a significant difference found between 84 mM glucose and 84 mM glucose combined with forskolin (*p* < 0.001). In addition, the amount of insulin secreted at 84 mM glucose with forskolin was significantly higher than the amount of insulin secreted at 24 mM glucose with forskolin (*p* < 0.01). Overall, the cAMP pathway has the capacity to dramatically increase insulin secretion, even at extreme glucose concentrations.

MDL-12,330A (MDL), an adenylyl cyclase inhibitor, was used to determine if reducing cAMP by inhibiting adenylyl cyclase could block the additional insulin secretion in extreme hyperglycemic conditions. MDL appeared to partially inhibit insulin secretion in 84 mM glucose. The significantly higher levels of insulin secretion in 84 mM glucose compared to 24 mM glucose were not significant with MDL added to 84 mM glucose (Figure 5B). Importantly, MDL did not reduce insulin secretion in 24 mM glucose compared to 24 mM glucose alone, indicating that the inhibitory effects of MDL on adenylyl cyclase occur only in extremely high glucose conditions when adenylyl cyclase is likely contributing more to overall insulin secretion.

Pancreatic islets were next stimulated with exendin-4, a GLP-1 agonist, to examine the possible contributions of incretin pathways on insulin secretion in extreme glucose conditions. There was a significant increase in insulin secretion with exendin added to 24 mM glucose compared to 24 mM glucose alone (*p* < 0.05). However, when exendin was tested with 84 mM glucose, there was no significant difference between 84 mM glucose and 84 mM glucose with exendin (Figure 5C). This indicates that insulin secretion, due to incretin effects, continues to increase between 24 mM and 84 mM glucose, revealing possible incretin involvement in the amplification pathway at extreme glucose concentrations.

## 3. Discussion

### 3.1. Maximum Glucose Concentrations for Glucose-Stimulated Insulin Secretion Are Much Higher Than Previously Reported

Our study demonstrates a surprisingly high capacity of islets to maintain stimulus-secretion coupling in glucose concentrations far exceeding what is considered normal for both mice and humans. We showed that insulin secretion doubled from 24 mM to 84 mM glucose, while calcium levels were unchanged over the same range. This suggests that insulin secretion occurs through the amplifying pathway, which can contribute 50% or more to insulin secretion when calcium is saturated [22]. In addition, our findings suggest that there is still much to be learned about the mechanisms and limits of the amplifying pathway of glucose-stimulated insulin secretion.

### 3.2. High Glucose Leads to Increased cAMP via Different Potential Mechanisms

Insulin secretion stimulated by cAMP can be thought of in two independent routes, the secretion that is dependent on calcium changes and that which is stimulated from cellular glucose metabolism [16]. Once cAMP accumulates, intracellular calcium rises through increased L-type-calcium-channel activity [23,24] and through release from intracellular stores [25]. Although cAMP thus has the capacity to increase intracellular calcium, our results reveal that intracellular calcium is already saturated in 24 mM glucose, so any additional stimulation of insulin secretion would likely not involve changes in calcium-dependent pathways. This study demonstrates that extremely high glucose levels can increase insulin secretion independently of the triggering pathway.

**Figure 6:**
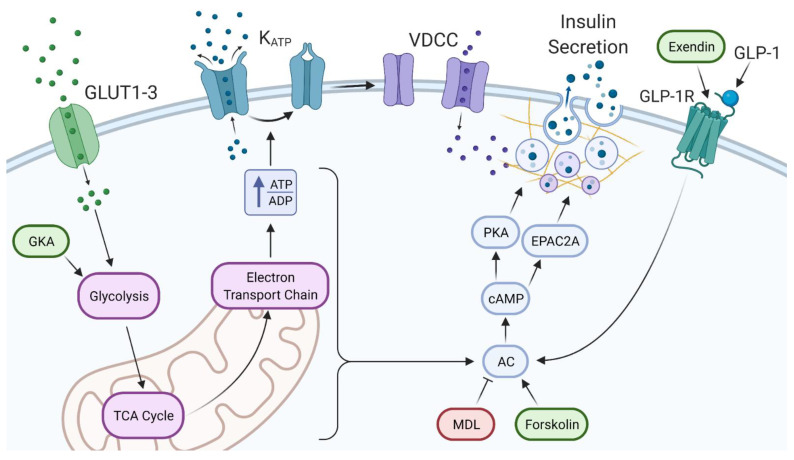
The triggering and amplifying pathways of insulin secretion in extreme glucose. (Left) Glucose enters the beta cell and is metabolized, causing an increase in the ATP to ADP ratio. ATP-sensitive potassium channels (KATP) close, causing membrane depolarization, the opening of voltage-dependent calcium channels (VDCC), and insulin secretion. (Right) GLP-1 from alpha cells or exendin stimulate beta-cell GLP-1 receptors which stimulate AC. Additionally, various metabolites produced from glucose metabolism can stimulate AC. Increased production of cAMP stimulates PKA and EPAC2A, causing increased insulin secretion by various mechanisms. Ovals indicate points of stimulation (green) or inhibition (red) for pharmacological agents used in these studies.

It is understood that increased glucose leads to an increase in cAMP [26]. Our data show that glucose continues to dose-dependently increase rates of glycolysis, as shown by a near doubling of insulin secretion between 24 and 84 mM glucose. As shown in Figure 6, the effects of extreme glucose can be reproduced with GKA in 24 mM glucose. This indicates that glycolytically driven metabolite formation is responsible for a large portion of insulin secreted above 24 mM glucose. Additionally, it should be noted that in Figure 6, forskolin and MDL act directly on adenylyl cyclase to alter cAMP levels. Forskolin, which acts to increase adenylyl cyclase, showed a huge potential for stimulating insulin secretion at high glucose concentrations. With that, MDL was able to partially block the insulin released in extremely high glucose conditions. MDL may not have fully reduced the insulin secreted in 84 mM glucose back down to 24 mM levels as expected, since other pathways independent of cAMP are likely working synergistically to amplify insulin secretion. Regardless, there are several ways in which increased glucose metabolism can augment insulin secretion through cAMP that do not require changes in intracellular calcium.

Protein kinase A (PKA) has a permissive role in increasing insulin secretion that has been shown to be glucose dependent [26]. This should be no surprise, since cAMP/PKA pathways have been long explored as targets for potential diabetes therapy [27]. As shown in Figure 6, cAMP activates downstream effectors PKA and guanine-nucleotide exchange protein (EPAC), which both lead to cAMP-mediated insulin secretion [28]. Focusing on the PKA pathway, glutamate derived from glucose through the malate aspartate shuttle is the specific signal underlying insulin secretion after being stimulated via cAMP [12]. It has been shown that the cAMP/PKA pathway potentiates the release of insulin via increased effectiveness of K_ATP_-channel-independent actions of glucose [29], which is consistent with our observations. Intracellular calcium is unaffected by PKA activation, and PKA effects on insulin secretion are mediated by the phosphorylation of various downstream proteins [30]. The mechanisms leading to the rise in insulin secretion are studied with electrophysiological and optical methods which monitor the movements and exocytosis of individual insulin granules [31,32]. Changes in the size of distinct granular pools, facilitation of granule recruitment from the pools to the plasma membrane, and the acceleration of the priming process that confers granules with release competence may all play a role as well as the involvement of SNARE complexes. Relating this back to our study, the PKA pathway stemming from the increase in cAMP ties to the amplification pathway via glucose metabolism and is most likely a key player in insulin release at such high glucose concentrations.

In addition to what we have shown, there are other glucose-associated shunts that lead to insulin secretion through the amplification pathway. Our data suggest that glycolysis itself maintains glucose dependence well above 24 mM glucose. Although, other than direct intermediates of glucose metabolism, there are potential excess-fuel detoxification pathways dealing with glycerol and free fatty acid formation and their extracellular release [32]. Free fatty acids play a role in insulin secretion by stimulating monoacylglycerol formation, whereas the inhibition of monoacylglycerol lipase activity decreases insulin secretion [33]. The aforementioned pathways may include the diversion of glucose carbons to triglycerides and cholesterol esters. Aspects relating to mitochondrial energy metabolism independent signals, including 1-monoglycerol, diacylglycerol, and malonyl-CoA are pieces of the amplification pathway to explore at high glucose levels. [33]. Future studies should investigate glycerol release and free fatty acids in extreme glucose conditions to further understand these intricate pathways.

### 3.3. Paracrine Effects of Extreme Glucose via Alpha Cells

Increasing glucose levels have been shown to lead to the induction of cAMP oscillations in both alpha and beta cells [34]. The alpha cells in pancreatic islets secrete GLP-1, which generally suppresses glucagon secretion [35]. Glucose-related glucagon secretion is observed in islets and reflects direct effects on alpha cells [29,30]. L-arginine can potently stimulate GLP-1 release in islets, and there is evidence that glucose may potentiate L-arginine-stimulated insulin secretion via PKA [36]. The relationship between glucose and GLP-1R is pivotal to understanding how glucose leads to an increase in cAMP.

It is known that glucose inhibits glucagon secretion by lowering cytoplasmic calcium in the alpha-cell; however, stimulation of glucagon at high glucose concentrations does not require an increase in intracellular calcium, and at higher glucose, glucagon secretion is actually stimulated. This paradoxical stimulation of glucagon release occurs around at least 25–30 mM [30,31]. In fact, high glucose has been shown to have a stimulatory effect on glucagon secretion possibly exceeding that of the inhibitory influence [37,38,39]. Looking at Figure 6, our studies demonstrate the connection of increasing glucose concentrations to increased GLP-1 and increased glucagon secreted by alpha cells. Our study suggests that the increase in incretins and glucagon from the alpha cells at high glucose concentrations could act on the beta cell to augment insulin secretion through the GLP-1 receptor pathway at these extreme levels.

### 3.4. Clinical Relevance

Diabetic patients have survived extreme glucose levels of over 100 mM [2,4,10]. Our study shows that insulin secretion occurs at extreme glucose levels, but as the concentration of glucose in the blood increases, insulin secretion in the higher glucose range does not keep to the same rate as in lower glucose concentrations, which is evidenced by the decrease in slope (Figure 1). However, this ability of beta cells to secrete insulin in these extremes of hyperglycemia is what distinguishes hyperosmolar hyperglycemic nonketoic syndrome from diabetic ketoacidosis. There is enough insulin present to prevent ketosis but not sufficient insulin to stimulate glucose utilization in target tissues (~10× as much insulin needed) [40]. We also observed that the extreme osmolarity increase associated with extreme hyperglycemia does not appear to negatively impact insulin secretion, at least in our in vitro studies in mouse islets. Thus, although individuals have survived, their bodies endured extreme stress during these instances. It should be noted that glucotoxicity has an effect on limiting the body’s ability to secrete insulin in extreme conditions, but this is considered a more chronic state than what we report.

Overall, we showed that insulin secretion from islets of both mice and human donors continues to increase in a dose-dependent manner to much higher glucose levels than previously thought. It is possible that novel pathways to insulin secretion could be identified only by stimulation in extremely high glucose. Once identified, it may be possible to develop novel therapeutics that could stimulate this secretory activity without requiring extremely high glucose.

### 3.5. Strengths and Limitations

An important strength of this study was the consistent observation in both murine and human islets in multiple trials which showed that the dose-dependent range of glucose-stimulated insulin secretion extends far higher than commonly thought. We further show that these increases rely on increases in glycolytic activity and cAMP, but not on changes in intracellular calcium. In addition, these studies show that osmolarity does not impact insulin secretion in vitro, which eliminates a potential confounding variable. Limitations in this study include the fact that isolated islets in vitro lack normal neural and humoral inputs found in vivo that can modulate function. Islets also lack the vasculature of their in vivo environment, which can impact how nutrients like glucose reach the islet. These are issues common to any in vitro study of pancreatic islets. Lastly, although our study shows an important role for cAMP in the insulin response to extreme glucose, many other factors of the amplification pathway could also be involved. Examining additional mechanisms will be the focus of future work.

## 4. Materials and Methods

### 4.1. Islet Sources and Isolation

Mouse islets were isolated from male CD-1 (Envigo, Indianapolis, IN, USA) mice ages 8–12 weeks, as previously described [41]. Briefly, pancreatic islets were isolated using collagenase-*p* digestion (Roche Diagnostics, Indianapolis, IN, USA) followed by centrifugation using Histopaque 1100 (Sigma-Aldrich, St. Louis, MO, USA). Islets were allowed to recover overnight in RPMI 1640 (Invitrogen, Carlsbad, CA, USA), supplemented with 10% fetal bovine serum and 1% penicillin/streptomycin before being used for experiments. All animal procedures were approved by the Ohio University Institutional Animal Care and Use Committee. Human islets from deidentified donors were obtained from the University of Alberta IsletCore and the University of Alberta/Alberta Health Services Clinical Islet Laboratory.

### 4.2. Calcium Imaging 

Fura-2 AM fluorescence imaging was utilized to measure intracellular calcium levels. Perifused solutions first passed through an inline heater to a temperature of 35+/−3 degrees Celsius into an open diamond bath imaging chamber (Warner Instruments, Cat: 64-0288) which was mounted using a stage adapter (Warner Instruments, Cat: 64-0298). Observation of islets was performed using a Hamamatsu ORCA-Flash4.0 digital camera (Hamamatsu Photonics K.K., Hamamatsu City, Japan, Model C11440-22CU) mounted on a BX51WIF fluorescence microscope with a 10X objective (Olympus, Tokyo, Japan). Excitation light was provided by a xenon burner supplied to the image field through a light pipe and filter wheel (Sutter Instrument Co., Novato, CA, USA, Model LB-LS/30) with a Lambda 10-3 Optical Controller (Sutter Instrument Co., Novato, CA, USA, Model LB10-3-1572). Images were taken sequentially with 340 nm and 380 nm excitation to produce each ratio from emitted light at 510 nm. Data were analyzed using cellSens Dimension 1.13 imaging software (Olympus, Tokyo, Japan) [42].

Islets were exposed to KRB containing 1 µM fura-2 AM and incubated for 30 min. They then were transferred onto the calcium scope. The fura-2 signal was recorded for two different experimental protocols. Protocol 1: 3 mM glucose (G) for 5 min, 24 G for 15 min, and 84 G for 15 min. Protocol 2: 3 mM glucose (G) for 5 min, 24 G for 15 min, 24 G containing 250 µM tolbutamide for 15 min, and back to 3 G for 25 min.

### 4.3. Insulin Secretion

To study insulin secretion, glucose-stimulated insulin secretion (GSIS) assays were performed. All GSIS used 12-well plates with 20 islets per well in 1 mL of KRB solution. Islets were size matched to aid in normalization as discussed in [43]. Briefly, islets were placed in 0 mM glucose for one hour, then transferred to the experimental conditions for an additional hour. Supernatants were collected from experimental conditions of 0, 12, 24, 36, 48, 60, 72, 84, and 144 mM. Using the same approach, we studied additional conditions using osmolarity-matched solutions at ~425 mOsm. Osmolarity was measured with Wescor Vapor Pressure Osmometer (Model 5520). Specific stimulators or inhibitors were placed into 24 G and 84 G to examine effects of exendin (10 nM, Sigma Aldrich, St. Louis, MO, USA, [18]), forskolin (10 µM, Sigma Aldrich, St. Louis, MO, USA, [44]), MDL-12330A (10 µM, Sigma Aldrich, St. Louis, MO, USA, [45]), and Ro-28-1675 (500 nM, Axon Medchem (Reston, VA, USA)). Insulin secretion was measured using mouse (Cat#80-INSMSU-E10) and human (Cat#80-INSHU-E01.1) ELISA following the manufacturer’s directions (ALPCO, Salem, NH, USA). Intra-assay variability was kept to below 15% for all studies. D-mannitol (Sigma-Aldrich, St. Louis, MO, USA) was used to balance the osmolarity of Modified KRB solutions to ~425 mOsm.

### 4.4. Cell Death Quantification

Islets were incubated in a glucose solution for 48 h in standard RPMI media supplemented 10% fetal bovine serum and 1% penicillin/streptomycin. Twenty islets were placed in each well per treatment in a 12-well plate. The 12-well plate contained the following treatments. Standard RPMI media, RPMI media+144 mM mannitol, RPMI media+60 mM glucose+84 mM mannitol, and finally RPMI+144 mM glucose. Cell death was measured with propidium iodide (Sigma-Aldrich, St. Louis, MO, USA) and annexin V (Invitrogen, Carlsbad, CA, USA) staining. Apoptosis was measured using annexin V (488 nm excitation/525 nm emission), which detects phosphatidylserine when it is exposed to the outer leaflet of the plasma membrane during apoptosis [46]. Propidium iodide (535 nm excitation/620 nm emission), which is a cell exclusion dye, was used to detect generalized cell death. Regions of interest were drawn around islets to measure fluorescence intensity per islet for each individual islet normalized to surface area. These techniques have been used in previous publications [47,48] including in comparison to other methods to measure cell death [49].

### 4.5. Statistical Analysis

Statistical analysis was performed using R Statistical Computing Software. Data are expressed as the mean ± standard error of the mean. Data were tested for normality using Shapiro-Wilk test and for equal variance using Levene’s test. Henze-Zirkler’s multivariate normality test was used for the insulin correlation data. All comparisons were analyzed using two-tailed *t*-test for comparisons of two groups or one-way ANOVA with Tukey’s post hoc test for more than two groups. Differences between groups were considered significant at *p* < 0.05. Spearman’s rank correlation coefficient was used to analyze insulin secretion patterns in Figure 1.

### 4.6. Ethical Approval

All animal procedures were approved by the Ohio University Institutional Animal Care and Use Committee. Human islet isolation was approved by the Human Research Ethics Board at the University of Alberta (Pro00013094). All donors’ families gave informed consent for the use of pancreatic tissue in research. Donor information was deidentified prior to our acquisition.

## Figures and Tables

**Table 1:** Human islet donor information.

Source	Isolation ID	Purity	Donor Age	BMI	Height (m)	Weight (kg)	HbA1c	Sex	Diabetes	Cold Ischemia Time (h)	Donation Type
IsletCore, University of Alberta	R286	95%	41	20.4	1.80	66	5.2	M	No	13.5	Neurological
IsletCore, University of Alberta	R318	90%	54	20.5	1.85	70	5.0	M	No	16	Neurological
IsletCore, University of Alberta	R322	90%	44	23.2	1.58	58	4.9	F	No	11.5	Neurological
Clinical Islet Lab, University of Alberta	H#:2296	90%	52	29.2	1.82	96.8	5.2	F	No	4.5	Head trauma (fall)

## Data Availability

All data used to support the findings of this study are available from the corresponding author upon request.

## References

[1] Komatsu M., Takei M., Ishii H., Sato Y. (2013). Glucose-stimulated insulin secretion: A newer perspective. J. Diabetes Investig..

[2] Gupta A., Rohrscheib M., Tzamaloukas A.H. (2008). Extreme hyperglycemia with ketoacidosis and hyperkalemia in a patient on chronic hemodialysis. Hemodial. Int..

[3] Kharroubi A.T., Darwish H.M. (2015). Diabetes mellitus: The epidemic of the century. World J. Diabetes.

[4] Ahlsson F., Gedeborg R., Hesselager G., Tuvemo T., Enblad P. (2004). Treatment of extreme hyperglycemia monitored with intracerebral microdialysis. Pediatr. Crit. Care Med..

[5] Alcazar O., Buchwald P. (2019). Concentration-Dependency and Time Profile of Insulin Secretion: Dynamic Perifusion Studies with Human and Murine Islets. Front. Endocrinol.

[6] Druet C., Tubiana-Rufi N., Chevenne D., Rigal O., Polak M., Levy-Marchal C. (2006). Characterization of Insulin Secretion and Resistance in Type 2 Diabetes of Adolescents. J. Clin. Endocrinol. Metab..

[7] Johnson D., Shepherd R.M., Gill D., Gorman T., Smith D.M., Dunne M.J. (2007). Glucose-dependent modulation of insulin secretion and intracellular calcium ions by GKA50, a glucokinase activator. Diabetes.

[8] Ramachandran K., Peng X., Bokvist K., Stehno-Bittel L. (2014). Assessment of re-aggregated human pancreatic islets for secondary drug screening. Br. J. Pharmacol..

[9] Lewandowski S.L., Cardone R.L., Foster H.R., Ho T., Potapenko E., Poudel C., VanDeusen H.R., Sdao S.M., Alves T.C., Zhao X. (2020). Pyruvate Kinase Controls Signal Strength in the Insulin Secretory Pathway. Cell Metab..

[10] Gopalakrishnan M., Manappallil R.G., Ramdas D., Jayaraj J. (2017). The survival story of a diabetic ketoacidosis patient with blood sugar levels of 1985 mg/dL. Asian J. Med Sci..

[11] Highest Blood Sugar Level. Guinness World Records. https://www.guinnessworldrecords.com/world-records/highest-blood-sugar-level/.

[12] Gheni G., Ogura M., Iwasaki M., Yokoi N., Minami K., Nakayama Y., Harada K., Hastoy B., Wu X., Takahashi H. (2014). Glutamate Acts as a Key Signal Linking Glucose Metabolism to Incretin/cAMP Action to Amplify Insulin Secretion. Cell Rep..

[13] Henquin J.C. (2000). Triggering and amplifying pathways of regulation of insulin secretion by glucose. Diabetes.

[14] Kalwat M.A., Cobb M.H. (2017). Mechanisms of the amplifying pathway of insulin secretion in the β cell. Pharmacol. Ther..

[15] Rustenbeck I., Schulze T., Morsi M., Alshafei M., Panten U. (2021). What Is the Metabolic Amplification of Insulin Secretion and Is It (Still) Relevant?. Metabolites.

[16] Tengholm A. (2012). Cyclic AMP dynamics in the pancreatic β-cell. Upsala J. Med. Sci..

[17] Evans-Molina C., Mirmira R.G. (2013). Achieving ‘PeaK-A’ Insulin Secretion. Diabetes.

[18] Peyot M.-L., Gray J.P., Lamontagne J., Smith P.J., Holz G.G., Madiraju S.M., Prentki M., Heart E. (2009). Glucagon-like peptide-1 induced signaling and insulin secretion do not drive fuel and energy metabolism in primary rodent pancreatic β-cells. PLoS ONE.

[19] Tenny S., Patel R., Thorell W. (2021). Mannitolin. StatPearls.

[20] Lee B., Jonas J.C., Weir G.C., Laychock S.G. (1999). Glucose regulates expression of inositol 1,4,5-trisphosphate receptor isoforms in isolated rat pancreatic islets. Endocrinology.

[21] Jonkers F.C., Guiot Y., Rahier J., Henquin J.-C. (2001). Tolbutamide stimulation of pancreatic β-cells involves both cell recruitment and increase in the individual Ca^2+^ response. Br. J. Pharmacol..

[22] Henquin J.C. (2009). Regulation of insulin secretion: A matter of phase control and amplitude modulation. Diabetologia.

[23] Henquin J.C., Meissner H.P. (1984). The ionic, electrical, and secretory effects of endogenous cyclic adenosine monophosphate in mouse pancreatic B cells: Studies with forskolin. Endocrinology.

[24] Yada T., Itoh K., Nakata M. (1993). Glucagon-like peptide-1-(7-36)amide and a rise in cyclic adenosine 3′,5′-monophosphate increase cytosolic free Ca^2+^ in rat pancreatic beta-cells by enhancing Ca^2+^ channel activity. Endocrinology.

[25] Liu Y.J., Grapengiesser E., Gylfe E., Hellman B. (1996). Crosstalk between the cAMP and inositol trisphosphate-signalling pathways in pancreatic beta-cells. Arch. Biochem. Biophys..

[26] Chepurny O.G., Kelley G.G., Dzhura I., Leech C.A., Roe M.W., Dzhura E., Li X., Schwede F., Genieser H.G., Holz G.G. (2009). PKA-dependent potentiation of glucose-stimulated insulin secretion by Epac activator 8-pCPT-2′-O-Me-cAMP-AM in human islets of Langerhans. Am. J. Physiol. Endocrinol. Metab..

[27] Yang H., Yang L. (2016). Targeting cAMP/PKA pathway for glycemic control and type 2 diabetes therapy. J. Mol. Endocrinol..

[28] Seino S., Shibasaki T. (2005). PKA-dependent and PKA-independent pathways for cAMP-regulated exocytosis. Physiol. Rev..

[29] Yajima H., Komatsu M., Schermerhorn T., Aizawa T., Kaneko T., Nagai M., Sharp G.W., Hashizume K. (1999). cAMP enhances insulin secretion by an action on the ATP-sensitive K+ channel-independent pathway of glucose signaling in rat pancreatic islets. Diabetes.

[30] Kaihara K.A., Dickson L.M., Jacobson D.A., Tamarina N., Roe M.W., Philipson L.H., Wicksteed B. (2013). β-Cell–Specific Protein Kinase A Activation Enhances the Efficiency of Glucose Control by Increasing Acute-Phase Insulin Secretion. Diabetes.

[31] Gaisano H.Y. (2014). Here come the newcomer granules, better late than never. Trends Endocrinol. Metab..

[32] Henquin J.-C., Nenquin M. (2014). Activators of PKA and Epac Distinctly Influence Insulin Secretion and Cytosolic Ca^2+^ in Female Mouse Islets Stimulated by Glucose and Tolbutamide. Endocrinology.

[33] Lamontagne J., Al-Mass A., Nolan C.J., Corkey B.E., Madiraju S.M., Joly E., Prentki M. (2017). Identification of the signals for glucose-induced insulin secretion in INS1 (832/13) β-cells using metformin-induced metabolic deceleration as a model. J. Biol. Chem..

[34] Tian G., Sandler S., Gylfe E., Tengholm A. (2011). Glucose- and hormone-induced cAMP oscillations in α- and β-cells within intact pancreatic islets. Diabetes.

[35] Liu P., Song J., Liu H., Yan F., He T., Wang L., Shen H., Hou X., Chen L. (2018). Insulin regulates glucagon-like peptide-1 secretion by pancreatic alpha cells. Endocrine.

[36] Thams P., Capito K. (1999). L-arginine stimulation of glucose-induced insulin secretion through membrane depolarization and independent of nitric oxide. Eur. J. Endocrinol..

[37] Salehi A., Vieira E., Gylfe E. (2006). Paradoxical stimulation of glucagon secretion by high glucose concentrations. Diabetes.

[38] Yu Q., Shuai H., Ahooghalandari P., Gylfe E., Tengholm A. (2019). Glucose controls glucagon secretion by directly modulating cAMP in alpha cells. Diabetologia.

[39] Gylfe E., Gilon P. (2014). Glucose regulation of glucagon secretion. Diabetes Res. Clin. Pract..

[40] Gosmanov A.R., Gosmanova E.O., Kitabchi A.E., Feingold K.R., Anawalt B., Boyce A., Chrousos G., de Herder W.W., Dhatariya K., Dungan K., Grossman A., Hershman J.M., Hofland J. (2000). Hyperglycemic Crises: Diabetic Ketoacidosis (DKA), and Hyperglycemic Hyperosmolar State (HHS). Endotext.

[41] Corbin K.L., West H.L., Brodsky S., Whitticar N.B., Koch W.J., Nunemaker C.S. (2021). A Practical Guide to Rodent Islet Isolation and Assessment Revisited. Biol. Proced. Online.

[42] Whitticar N.B., Strahler E.W., Rajan P., Kaya S., Nunemaker C.S. (2016). An Automated Perifusion System for Modifying Cell Culture Conditions over Time. Biol. Proced. Online.

[43] Slepchenko K.G., Corbin K.L., Nunemaker C.S. (2019). Comparing methods to normalize insulin secretion shows the process may not be needed. J. Endocrinol..

[44] Ammon H.P.T., Muller A.B. (1984). Effect of forskolin on islet cyclic AMP, insulin secretion, blood glucose and intravenous glucose tolerance in rats. Naunyn-Schmiedeberg’s Arch. Pharmacol..

[45] Li X., Guo Q., Gao J., Yang J., Zhang W., Liang Y., Wu D., Liu Y., Weng J., Li Q. (2013). The Adenylyl Cyclase Inhibitor MDL-12,330A Potentiates Insulin Secretion via Blockade of Voltage-Dependent K+ Channels in Pancreatic Beta Cells. PLoS ONE.

[46] Baskić D., Popović S., Ristić P., Arsenijević N.N. (2006). Analysis of cycloheximide-induced apoptosis in human leukocytes: Fluorescence microscopy using annexin V/propidium iodide versus acridin orange/ethidium bromide. Cell Biol. Int..

[47] Dula S.B., Jecmenica M., Wu R., Jahanshahi P., Verrilli G.M., Carter J.D., Brayman K.L., Nunemaker C.S. (2010). Evidence that low-grade systemic inflammation can induce islet dysfunction as measured by impaired calcium handling. Cell Calcium.

[48] Gelin L., Li J., Corbin K.L., Jahan I., Nunemaker C.S. (2018). Metformin Inhibits Mouse Islet Insulin Secretion and Alters Intracellular Calcium in a Concentration-Dependent and Duration-Dependent Manner near the Circulating Range. J. Diabetes Res..

[49] O’Neill C.M., Lu C., Corbin K.L., Sharma P.R., Dula S.B., Carter J.D., Ramadan J.W., Xin W., Lee J.K., Nunemaker C.S. (2013). Circulating Levels of IL-1B+IL-6 Cause ER Stress and Dysfunction in Islets from Prediabetic Male Mice. Endocrinology.

